# 13623 Daily relationship between social connectedness and health behaviors among dementia family caregivers

**DOI:** 10.1017/cts.2021.651

**Published:** 2021-03-31

**Authors:** Kylie Meyer, Sara Masoud, Ashlie Glassner, Kevin Hamilton, Ariel Chinea, Darpan Patel, Jing Wang, Carole White

**Affiliations:** University of Texas Health Sciences at San Antonio

## Abstract

ABSTRACT IMPACT: Knowledge of which aspects of social connectedness most strongly associate with caregiver health and health behaviors can inform intervention targets to improve caregiver health OBJECTIVES/GOALS: Stressed dementia caregivers are at risk of poor health. Social connectedness may reduce adverse health effects, yet it is unknown about which aspects relate most strongly to health. This is a barrier to intervention development. Our study identifies aspects of social connectedness most strongly associate with caregivers’ daily health behaviors. METHODS/STUDY POPULATION: Data. Enrolled spousal caregivers completed 14 consecutive days of online surveys. Measures. We examined multiple health behaviors each day, which included: 1) number of occurrences of 3 potential binge-eating behaviors (range 0 to 30), 2) whether participants engaged in at least 30 minutes of physical activity, and 3) perceived sleep quality, rated 1 (very bad) to 5 (very good). We also examined a count of health symptoms caregivers experienced (e.g., backache; range: 0 to 7). Measures of social connectedness included: spousal emotional support, perceived spousal appreciation, emotional support from any source, and loneliness. Analysis. We applied bivariate multi-level mixed effects models to examine the association between each aspect of social connectedness and health behaviors day-to-day. RESULTS/ANTICIPATED RESULTS: Since November 2020, 5 of N=40 participants were enrolled, of whom 3 had completed all diary surveys. Participants were women ages 59 to 73, and included 4 non-Hispanic white and 1 Hispanic caregivers. Data included 51 days of surveys (93% adherence). No differences in eating behaviors nor physical activity according to social connectedness were found. Emotional support from any source was positively associated with sleep quality (B= 0.20; SE=0.08; p-value 0.015). On days when caregivers indicated their spouse appreciated them ‘A lot’ versus ‘Not at all,’ sleep quality was marginally better (B=0.73, SE=0.42; p-value-0.08). Finally, days when caregivers felt lonely at least ‘Some of the time’ versus ‘Not at all’ were associated with experiencing more adverse health symptoms (B=1.54; SE=0.58; p-value<0.001). DISCUSSION/SIGNIFICANCE OF FINDINGS: Improved emotional support from any source may support better sleep quality among caregiving spouses, while loneliness appears to contribute to more adverse health symptoms. Findings, if confirmed, can be translated to develop intervention programs that target loneliness and perceived emotional support among caregivers.

